# Reduction in Responding for Sucrose and Cocaine Reinforcement by Disruption of Memory Reconsolidation^1^,^2^,^3^

**DOI:** 10.1523/ENEURO.0009-15.2015

**Published:** 2015-03-30

**Authors:** Marc T. J. Exton-McGuinness, Jonathan L. C. Lee

**Affiliations:** School of Psychology, University of Birmingham, B15 2TT, United Kingdom

**Keywords:** cocaine, dopamine, NMDAR, nucleus accumbens, reconsolidation, sucrose

## Abstract

Stored memories are dynamic and, when reactivated, can undergo a process of destabilization and reconsolidation to update them with new information. Reconsolidation has been shown for a variety of experimental settings; most recently for well-learned instrumental memories, a class of memory previously thought not to undergo reconsolidation. Here we tested, in rats, whether a weakly-trained lever-pressing memory destabilized following a shift in reinforcement contingency. We show that lever-pressing memory for both sucrose and cocaine reinforcement destabilized under appropriate conditions, and that the reconsolidation of this memory was impaired by systemic administration of the NMDA receptor (NMDAR) antagonist [5R,10S]-[+]-5-methyl-10,1-dihydro-5*H*-dibenzo[*a*,*d*]cyclohepten-5,10-imine (MK-801). We went on to investigate the potential role of the nucleus accumbens (NAc) in the reconsolidation of sucrose-reinforced instrumental memories, showing that co-infusion of the NMDAR antagonist 2-amino-5-phosphonopentanoic acid (AP-5) and the dopamine-1 receptor (D1R) antagonist 7-chloro-3-methyl-1-phenyl-1,2,4,5-tetrahydro-3-benzazepin-8-ol (SCH23390) into the NAc prior to memory reactivation impaired reconsolidation; however, there was no effect when these drugs were infused alone. Further investigation of this effect suggests the combined infusion disrupted the reconsolidation of pavlovian components of memory, and we hypothesize that coactivation of accumbal D1Rs and NMDARs may contribute to both the destabilization and reconsolidation of appetitive memory. Our work demonstrates that weakly-trained instrumental memories undergo reconsolidation under similar parameters to well-trained ones, and also suggests that receptor coactivation in the NAc may contribute to memory destabilization. Furthermore, it provides an important demonstration of the therapeutic potential of reconsolidation-based treatments that target the instrumental components of memory in maladaptive drug seeking.

## Abstract (precis)

This research adds to the growing body of evidence that instrumental memories (memories of interactions with the world) undergo reconsolidation, a class of memory previously not thought to undergo reconsolidation. Furthermore, we suggest that there may be a role for coactivation of accumbal D1Rs and NMDARs in the destabilization and reconsolidation of appetitive memory.

## Significance Statement

This research adds to the growing body of evidence that instrumental memories (memories of interactions with the world) undergo reconsolidation, a class of memory previously not thought to undergo reconsolidation. Furthermore, we suggest that there may be a role for coactivation of accumbal D1Rs and NMDARs in the destabilization and reconsolidation of appetitive memory. Our work also extends to include reconsolidation disruption of responding for cocaine self-administration. This provides proof of principle that impairing the reconsolidation of instrumental memory can diminish the instrumental components of drug seeking, and demonstrates the potential viability of reconsolidation-based therapies for maladaptive memory disorders.

## Introduction

Following an initial phase of consolidation, memories exist in a stable state, resistant to amnesic intervention (McGaugh, 2000). Memories are not fixed, however, and can be destabilized, rendering them labile to be updated with new information (Lee, 2009). In order to persist following destabilization, memories must undergo a process of reconsolidation that returns them to their stable form (Nader, 2003). Reconsolidation has been demonstrated for nearly all types of memory (for review, see Reichelt and Lee, 2013) and its initiation (via destabilization) appears to require a prediction error (Sevenster et al., 2013).

A notable cluster of negative findings within the field of reconsolidation have concerned instrumental memories, raising questions over whether reconsolidation is a universal process for memory persistence. Early studies observed that the instrumental components of sucrose (Hernandez and Kelley, 2004), saccharine (Mierzejewski et al., 2009), and cocaine (Brown et al., 2008) self-administration did not appear to undergo reconsolidation, although pavlovian memories associated with these behaviours did (Lee et al., 2006a; Milton et al., 2008b). Recently, instrumental memory underpinning well-learned sucrose seeking was shown to undergo reconsolidation (Exton-McGuinness et al., 2014b), and subsequently lever pressing for nicotine was also shown to undergo reconsolidation (Tedesco et al., 2014; although this result may not represent reconsolidation of the instrumental component of memory, see Exton-McGuinness et al., 2014a).

We first sought to investigate whether a weakly-trained instrumental memory would destabilize following a change in reinforcement contingency, from a fixed to a variable ratio schedule, as was recently demonstrated to be the case in a well-trained setting (Exton-McGuinness et al., 2014b). While it is an obvious prediction that both weakly and well-learned memories should be destabilized according to similar principles, it is well acknowledged that instrumental behaviors become automated with overtraining (Dickinson, 1985), becoming reliant on neural systems that are distinct from those used early on in training (Balleine and O’Doherty, 2010). Furthermore, older (Suzuki et al., 2004) and more extensively trained memories (Suzuki et al., 2004; Reichelt and Lee, 2012) generally require different reactivation parameters in order to destabilize (typically longer or more frequent stimulus presentation) compared to those that are sufficient following a more limited training regimen. These more extreme reactivation parameters could lead to extinction learning in which responding is suppressed by a new inhibitory memory (Bouton, 2002), rather than reconsolidation whereby the original memory is updated, when used with a younger, weaker memory (Reichelt and Lee, 2012). Thus, we elected to use a lesser shift in contingency to destabilize weakly-trained lever-pressing memory than was used previously in a well-trained setting (Exton-McGuinness et al., 2014b).

We also explored whether brief non-reinforced or training reactivations could destabilize lever-pressing memory. These additional reactivation parameters also acted to test the necessity for reinforcer presentation during reactivation, as the presence and consumption of the reinforcer may provide both external and internal stimuli, which contribute to memory destabilization (Milekic et al., 2006; Valjent et al., 2006; Brown et al., 2008). The efficacy of the reactivation conditions to destabilize instrumental memory and initiate reconsolidation was initially verified using systemic injections of the NMDAR antagonist MK-801, shown previously to disrupt reconsolidation of instrumental memory (Exton-McGuinness et al., 2014b).

We then progressed to intra-NAc infusions of AP-5 and SCH23390 in order to assess any potential role for local activation of D1Rs and NMDARs in the reconsolidation of instrumental memories, as coactivation of these receptors in the NAc is implicated in the acquisition of lever pressing (Smith-Roe and Kelley, 2000). We also infused MK-801 to determine whether the NAc was a central locus of action for systemic treatment. The NAc has been strongly implicated in mediating reward-seeking behaviors and is a key hub in the reward circuitry disrupted by addictive drugs such as cocaine (Robbins and Everitt, 1996; Loweth et al., 2014).

Disruption of reconsolidation may offer a novel therapeutic intervention for maladaptive memories, such as post-traumatic stress disorder (Pitman, 2011) and drug addiction (Milton, 2013). In order to demonstrate the translational benefit of disrupting instrumental memory reconsolidation, we tested whether lever pressing for cocaine self-administration would undergo reconsolidation following a shift in reward contingency. While a previous study showed MK-801 did not disrupt the reconsolidation of cocaine self-administration (Brown et al., 2008), we hypothesized this was due to inappropriate, or insufficient, reactivation parameters. Appetitive pavlovian memory for both sucrose and cocaine undergoes reconsolidation (Lee et al., 2006a; Milton et al., 2008b), and so it seemed logical that any successful impairment of sucrose-reinforced instrumental memory reconsolidation would translate to a cocaine self-administration setting.

## Materials and Methods

### Subjects

Subjects were 219 experimentally naïve adult male lister hooded rats (Charles River), aged 6-8 weeks (median 6 weeks) and weighing 200-350 g (median 250 g) at the start of the experiment. Rats were housed in individually-ventilated cages of 4 at 21 °C on a 12 h light-dark cycle (lights on at 0700) in a specialist animal facility. Individually-ventilated cages contained aspen chip bedding, and environmental enrichment was available in the form of a Plexiglass tunnel. Experiments took place in a behavioral laboratory between 0800 and 1200. Rats in the sucrose studies were fed a restricted diet of 15 g chow per day for the duration of the behavioral procedures (this was supplemented by any sucrose rewards obtained during the study); weights were regularly recorded and assessed against an in-house growth chart. Rats in the cocaine study had freely available food. Water was freely available except during experimental procedures. At the end of the experiment, animals were humanely killed via a rising concentration of CO_2_; death was confirmed by cessation of heartbeat. All procedures were approved by a local ethical review committee and carried out in accordance with the United Kingdom 1986 Animals (Scientific Procedures) Act (PPLs 40/3205 & 70/7662).

### Surgical procedures

All surgeries were performed aseptically in accordance with the LASA guiding principles for aseptic surgery (LASA, 2010). Rats were anaesthetized using isoflurane (5% for induction, 2-3% for maintenance), and administered peri-operative buprenorphine. Post-surgery, rats were housed individually with Puracel bedding overnight before being rehoused with their cage mates the next morning. Their diet was also supplemented with the non-steroidal anti-inflammatory Carprofen for 2 d post-operatively. A minimum of 5 d recovery was allowed before experimental procedures began.

**Figure 1:**
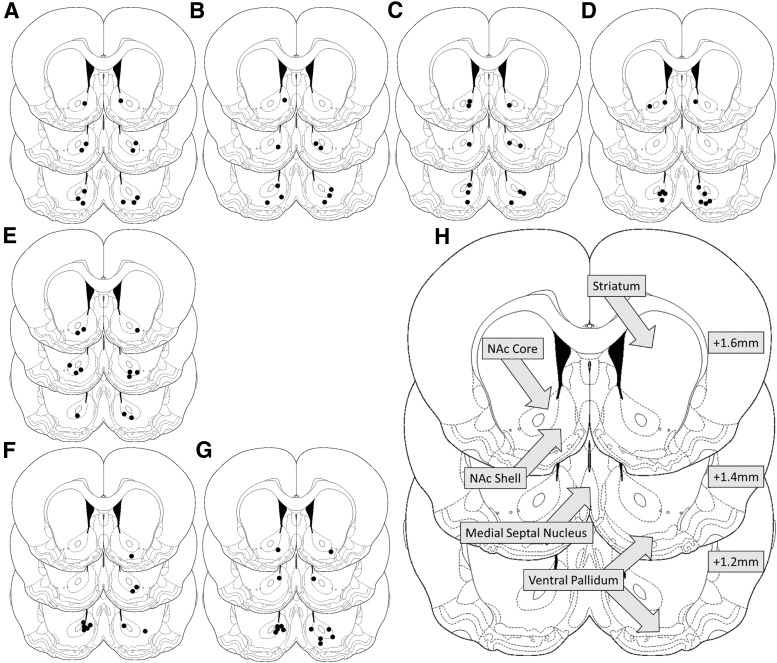
Schematic of the brain. Black dots indicate the location of injector tips for reactivated PBS (***A***), AP-5 (***B***), SCH23390 (***C***), AP-5/SCH23390 (***D***), MK-801 (***E***) infused groups and non-reactivated PBS (***F***), and AP-5/SCH23390 (***G***) controls. ***H***, Diagram showing location of notable brain regions surrounding the infusion site. Numbers (right) signify millimeters from bregma. All injectors were located within the NAc.

Eighty rats were implanted bilaterally with stainless steel cannulae (11 mm, 22 gauge; Coopers Needleworks). Cannulae were directed at the NAc region of the brain using a stereotaxic frame: AP +1.5 mm, ML ±1.8 mm from bregma, DV −1.8 mm from skull surface (Paxinos and Watson, 2009). Stainless steel stylets extending 1 mm past the end of the guide cannulae were inserted post-surgery in order to maintain patency until infusion. Prior to reactivation, stylets were removed and injectors (28 gauge; Plastics One) inserted into the guide cannulae, extending 6 mm past the end of the guide cannulae to a final DV −7.8 mm. PBS vehicle, MK-801, AP-5, SCH23390, or combined AP-5/SCH23390 (see Drugs, below) was infused into the NAc immediately prior to reactivation. At the end of the experiment, cannulated rats were killed, their brains extracted freshly and fixed in 4% paraformaldehyde. Brains were sectioned then stained using cresyl violet and the locations of injectors confirmed using light microscopy (Fig. 1).

For the cocaine study, 12 rats were catheterized by Charles River and an additional 20 were implanted with intravenous catheters based on previous literature (Di Ciano and Everitt, 2001; Lee et al., 2006a). Briefly, rats were implanted with a single catheter (Camcaths) in the right jugular vein aimed at the left vena cava. The mesh end of the catheter was sutured subcutaneously on the dorsum.

### Drugs

For the sucrose and cocaine self-administration studies, MK-801 (AbCam) was dissolved in sterile saline to a concentration of 0.1 mg/ml. Thirty minutes prior to the reactivation session, rats were administered intraperitoneally with 0.1 mg/kg of MK-801 or equivalent volume of saline vehicle. This dose of systemic MK-801 has previously been shown to disrupt instrumental memory reconsolidation (Exton-McGuinness et al., 2014b). Injections were carried out systematically by cage, randomly within each cage.

For intracerebral infusions, all drugs were dissolved in sterile PBS. AP-5 (AbCam), SCH23390 (RBI), and MK-801 were made to a concentration of 1 µg/0.5 µl. The combined AP-5/SCH23390 solution was made up to 0.1 µg/0.5 µl of each AP-5 and SCH23390. The choice of drugs and dosages were based on those shown previously to disrupt acquisition of lever pressing (Kelley et al., 1997; Smith-Roe and Kelley, 2000). Infusions were given systematically by cage, randomly within each cage. Immediately prior to the memory reactivation session, stylets were removed and injectors were inserted into the guide cannulae. Using a microdrive syringe pump (Harvard Apparatus), 0.5 µl of drug or PBS vehicle control was infused at a rate of 0.5 µl/min. Injectors were left in place for 1 min after the infusion to allow diffusion of the drug. Infusions were given immediately before, rather than after, reactivation as past work has shown that pre-session (Kelley et al., 1997; Smith-Roe and Kelley, 2000) but not post-session (Hernandez et al., 2005) infusions of AP-5 or SCH23390 into the NAc core impair instrumental acquisition. Moreover, the time window of vulnerability of reconsolidation to disruption appears to be more limited than for consolidation (Judge and Quartermain, 1982).

For cocaine self-administration, cocaine (Sigma-Aldrich) was dissolved in sterile saline to a concentration of 2.5 mg/ml; intravenous infusions of 0.1 ml over 5 s could be obtained during training and reactivation. Drug infusion dosage was based on previous literature (Di Ciano and Everitt, 2001).

### Behavioural apparatus

Training, memory reactivation, and testing sessions took place in eight operant boxes (MedAssociates) measuring 25 × 32 × 25.5 cm, each housed individually within a sound-attenuating chamber. The rear wall and door were made of Perspex, the other two walls of metal. The boxes contained a grid floor of 19 evenly spaced, stainless steel bars (4.8 mm diameter), underneath which was a removable tray. A nosepoke magazine was mounted on the right-hand wall into which the reward pellets could be delivered, flanked on either side by two retractable levers. The magazine contained an infrared detector that recorded magazine entries (nosepokes). The box was illuminated by a small houselight mounted on the upper left-hand wall, which came on at the start of each experimental session and switched off at the end. Two boxes were equipped with an infusion pump and drug delivery arm assembly (MedAssociates) for the intravenous delivery of cocaine. The operant boxes were run from a local computer using specialized computer software (MedAssociates), which also recorded behavioral responses (lever presses and nosepokes).

### Training procedures

#### Sucrose study

Rats were initially trained to collect 45 mg sucrose reward pellets (TestDiet) from the magazine. Pellets were delivered at random intervals (mean 60 s) for 15 min. This pre-training facilitated instrumental learning over the limited training schedule. Instrumental training began immediately after the pre-training session. On the first training day, a single lever was extended into the box and delivered a sucrose pellet into the magazine when pressed, on a fixed-ratio (FR1; one lever press delivers one pellet) schedule; responses on the lever had no other programmed consequence, the lever did not retract and remained extended throughout the session and no discrete stimuli were presented at any point during training. A maximum of 30 pellets could be obtained; the session ended when the maximum number of pellets had been obtained or 30 min elapsed. Rats received a second 30 min training session the next day with a maximum of 60 pellets obtainable and this marked the end of the training phase. Rats were injected systemically with MK-801 30 min prior to the memory reactivation session.

#### Intra-accumbans study

Behavioral procedures were carried out as in the sucrose study. Rats were implanted with bilateral cannualae aimed at the NAc (see Surgical procedures, above) and drugs were infused immediately before reactivation.

#### Cocaine study

Prior to each training or reactivation session, the implanted intravenous catheter (see Surgical procedures, above) was connected to the infusion arm. For the pre-operated rats, this was achieved using a vascular access harness and tether (Instech). For the rats catheterized in-house, the catheter was connected directly to the spring tether (Camcaths). A single lever was extended into the chamber at the start of each session. Rats were trained to lever press for cocaine on an FR1 schedule. A maximum of 30 intravenous cocaine infusions could be obtained on the first training day and 60 on the second; each session lasted a maximum of 2 h. After each infusion, there was an enforced time-out of 20 s before the next infusion could be obtained. The lever retracted for the duration of the time-out period. Rats did not receive any pre-training for the cocaine study, nor were any discrete reward-paired stimuli presented. Nosepokes had no programmed consequence. Rats were injected systemically with MK-801 30 min prior to the memory reactivation session.

### Reactivation procedures

In the systemic studies, a variety of behavioral conditions were tested for their efficacy to cause memory destabilization.

#### VR5 reactivation

A variable number of lever presses (mean: 5, range: 1-9) were required to obtain a reward. A maximum of 20 reinforcements could be obtained. The variable-ratio (VR5; one pellet is delivered following a mean of five lever presses) reactivation was also used for the intracerebral and cocaine studies. For sucrose studies, the session length was 20 min. For the cocaine study, the lever retracted for 20 s, as in training, and the session length was 30 min. This reactivation was chosen based on a previous finding that a variable-ratio schedule destabilized a well-established instrumental memory (Exton-McGuinness et al., 2014b).

#### Non-reactivation controls

Additional rats were used, which received systemic injection of MK-801 or coinfusion of AP-5/SCH23390 (or appropriate vehicle control), but without a reactivation session. This provided an important control for determining whether reconsolidation had been disrupted; amnestic treatment in the absence of memory reactivation should be without effect.

#### Non-reinforced reactivation

A very brief non-reinforced session lasted only 2 min; no rewards were delivered during this session. Brief non-reinforced sessions have been frequently used to destabilize pavlovian memories in past studies (Lee et al., 2006b; Milton et al., 2008b). In a previous study of well-learned lever pressing for sucrose, a 5 min non-reinforced session did not destabilize the instrumental memory (Exton-McGuinness et al., 2014b); however, it may be that a shorter non-reinforced reactivation could, as briefer sessions typically favor reconsolidation, while longer non-reinforced sessions usually lead to extinction (Flavell and Lee, 2013; Merlo et al., 2014), in which a new memory is formed that suppresses behavioral output (Bouton, 2002).

#### FR1 reactivation

A brief FR1 session was identical to that used in training but curtailed to a maximum of 20 pellets, with a maximum duration of 20 min. Training sessions have been shown to trigger memory destabilization, however, only when the memory is not well-learned (Rodriguez-Ortiz et al., 2005; Díaz-Mataix et al., 2013; Exton-McGuinness et al., 2014a), consistent with the hypothesis that reconsolidation serves memory updating (Lee, 2009). As the lever-pressing memory used in our study was only weakly trained, we hypothesized a training trial could also induce reconsolidation. This session also tested whether it was the variability of the VR5 reactivation contingency or simply the presence of the reinforcer that was the salient feature of the VR5 reactivation. The presence of the unconditioned stimulus is sometimes required to destabilize appetitive pavlovian memory (Milekic et al., 2006; Valjent et al., 2006), and furthermore the contingency of reinforcer presentation may also be an important factor in determining whether a memory will destabilize (Lee and Everitt, 2008a).

### Testing procedures

Instrumental performance was tested the day after drug treatment for all groups. Test sessions lasted 30 min and were performed in extinction. The lever was extended, no rewards were delivered, and the houselight remained on throughout the session. The lever did not retract during testing.

In the systemic sucrose study, one additional group of rats received their test session 3 h after the VR5 reactivation. This was done to assess any effect of the MK-801 on post-reactivation short-term memory (pr-STM). If pr-STM was disrupted by MK-801, then it might imply that MK-801 had effects other than disrupting long-term memory reconsolidation, which impacted on behavioral expression. Were the reconsolidation of long-term memory disrupted by MK-801, then there should typically be no effect on short-term memory expression.

### Statistical analysis

Data are represented as mean + SEM throughout. Results with *p* < 0.05 were deemed significant. Statistical analyses are summarized in Table 1 (superscript letters in the Results text indicate rows in the table). Observed power was calculated *post hoc* with G*Power 3.1 (Faul et al., 2007) using the size of the highest order effect at test.

**Table 1:** Summary of statistical analyses. Letters (left) refer to values within the Results section. Observed power was calculated using the highest order effect size at test.

	Data structure	Type of test	Observed power
a	Normally distributed	Repeated measures ANOVA	0.959
b	Normally distributed	One-way ANOVA	0.333
c	Normally distributed	Two-way ANOVA with *post hoc* simple effects	0.596
d	Normally distributed	Repeated-measures ANOVA with *post hoc* comparisons	0.077
e	Normally distributed	One-way ANOVA	0.056
f	Normally distributed	Two-way ANOVA	0.063
g	Normally distributed	Repeated-measures ANOVA	0.230
h	Normally distributed	One-way ANOVA	0.094
i	Normally distributed	Two-way ANOVA	0.094
j	Normally distributed	Repeated-measures ANOVA	0.878
k	Normally distributed	One-way ANOVA	0.347
l	Normally distributed	One-way ANOVA	0.347
m	Normally distributed	Repeated-measures ANOVA with *post hoc* comparisons	1.000
n	Normally distributed	One-way ANOVA	0.812
o	Normally distributed	One-way ANOVA	0.812
p	Normally distributed	Repeated-measures ANOVA	0.644
q	Normally distributed	One-way ANOVA	0.215
r	Normally distributed	One-way ANOVA	0.215
s	Normally distributed	Repeated-measures ANOVA	0.470
t	Normally distributed	One-way ANOVA	0.157
u	Normally distributed	Repeated-measures ANOVA	0.050
v	Normally distributed	One-way ANOVA	0.050
w	Normally distributed	One-way ANOVA	0.050
x	Normally distributed	Repeated-measures ANOVA with Bonferroni-corrected planned comparisons	1.000
y	Normally distributed	One-way ANOVA with Bonferroni-corrected planned comparisons	0.734
z	Normally distributed	One-way ANOVA with Bonferroni-corrected planned comparisons	0.734
aa	Normally distributed	Repeated-measures ANOVA with Bonferroni-corrected planned comparisons	0.981
bb	Normally distributed	One-way ANOVA with Bonferroni-corrected planned comparisons	0.450
cc	Normally distributed	One-way ANOVA with Bonferroni-corrected planned comparisons	0.450
dd	Normally distributed	Repeated-measures ANOVA with *post hoc* comparisons	0.996
ee	Normally distributed	Two-way ANOVA with *post hoc* simple effects	0.776
ff	Normally distributed	Repeated-measures ANOVA	0.964
gg	Normally distributed	Two-way ANOVA with *post hoc* simple effects	0.620
hh	Normally distributed	Repeated-measures ANOVA	0.995
ii	Normally distributed	One-way ANOVA	0.477
jj	Normally distributed	Two-way ANOVA with *post hoc* simple effects	0.768
kk	Normally distributed	Repeated-measures ANOVA	0.061
ll	Normally distributed	One-way ANOVA	0.053
mm	Normally distributed	Two-way ANOVA	0.055

Experimental groups were matched for number of lever presses made during training. Training data was analyzed using repeated-measures ANOVA with Training Day and Drug Group as factors in order to assess whether the task was learned and whether groups were similarly performing at the end of the training phase. Where appropriate, planned comparisons were performed on the second day of training in order to test for any pre-reactivation differences.

In the systemic experiments, reactivation and test sessions were analyzed separately using one-way ANOVA for rats given brief extinction or training sessions. For intracerebral infusion groups, reactivation and test data was compared to a single control using Bonferroni-corrected planned comparisons (effective *p* < 0.0125). Non-reactivated rats were compared to their respective reactivated counterparts using two-way ANOVA with Reactivation and Drug Treatment as factors. Similar analysis was performed on all sessions for magazine entries (nosepokes) in order to assess general activity. Differences in nosepokes may indicate differences in motivation, which may have impacted lever-pressing performance; however, it is important to note that, in the case of the sucrose studies, the magazine also acted as a reward location, which might have influenced the total number of nosepokes made.

In the sucrose-seeking studies, rats that failed to obtain at least 30 rewards on the second training day were excluded from the final analysis due to insufficient learning. For cocaine seeking, rats obtaining fewer than five rewards on the second day of training were excluded from further analysis. These criteria excluded 40 rats from the systemic sucrose experiments, 19 from the intracerebral study, and four from the cocaine study. Four rats were killed following surgical complications and did not start the experiment. Eleven rats had bent or blocked cannulae and were excluded from analysis as they did not receive bilateral infusions. An additional vehicle-treated rat was excluded from the analysis of the cocaine study as his data point lay more than 2 SDs from the group mean.

## Results

### Sucrose study

#### VR5 reactivation

We first tested whether a briefly-trained instrumental memory for sucrose reinforcement would destabilize following the VR5 reactivation, a session in which a variable number of lever presses were required to obtain a reward. If the memory was successfully destabilized, then its subsequent reconsolidation should be disrupted by systemic MK-801, leading to a reduction in long-term memory expression.

Treatment groups showed similar acquisition of lever pressing over the 2 d of training, confirming that rats learned to press the lever to acquire sucrose and that groups were well matched (data not shown; Training: *F*_(1,27)_ = 1244.0, *p* < 0.001^a^; Treatment: *F*_(1,27)_ = 0.02, *p* = 0.895^a^; Reactivation: *F*_(1,27)_ = 1.61, *p* = 0.215^a^; Treatment × Reactivation: *F*_(1,27)_ = 0.17, *p* = 0.684^a^; Training × Treatment: *F*_(1,27)_ = 2.48, *p* = 0.128^a^; Training × Reactivation: *F*_(1,27)_ = 0.64, *p* = 0.430^a^; Training × Treatment × Reactivation: *F*_(1,27)_ = 1.20, *p* = 0.282^a^). The day after training, rats were administered systemic MK-801 or saline control 30 min prior to the VR5 reactivation session. There was no significant difference in lever pressing between MK-801- (100.1 ± 5.2) and saline- (92.4 ± 9.7) treated groups during the VR5 memory reactivation session (*F*_(1,14)_ = 0.56, *p* = 0.468^b^).

**Figure 2:**
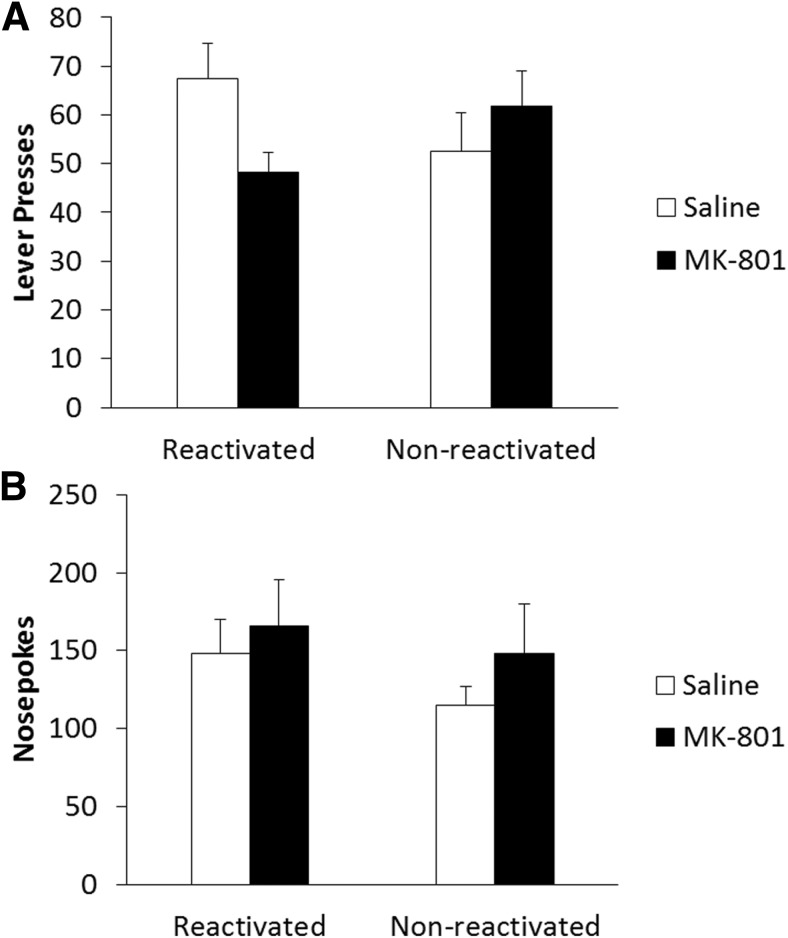
Systemic MK-801 impaired the reconsolidation of a weakly-learned lever-pressing memory for sucrose reinforcement following a shift to a VR5 schedule during reactivation. ***A***, MK-801 (*n* = 9) administered prior to the VR5 reactivation significantly impaired lever-pressing performance in a reactivation-dependent manner at test the next day. Performance of reactivated MK-801 rats was impaired compared to reactivated saline controls (*n* = 7); however, there was no significant difference between non-reactivated rats administered saline (*n* = 8) or MK-801 (*n* = 7). ***B***, MK-801 treatment had no significant effect on long-term nosepoking behavior regardless of reactivation. Data are represented as mean + SEM.

In a test of long-term memory, 24 h after drug administration and reactivation (Fig. 2*A*), there was a reactivation-dependent effect of MK-801 on lever pressing (Treatment × Reactivation: *F*_(1,27)_ = 4.53, *p* = 0.042^c^) with no significant main effects of treatment (*F*_(1,27)_ = 0.55, *p* = 0.464^c^) or reactivation (*F*_(1,27)_ = 0.01, *p* = 0.908^c^). Analysis of simple main effects showed significantly reduced lever pressing in MK-801-treated reactivated rats compared to reactivated saline controls (*F*_(1,14)_ = 6.02, *p* = 0.028^c^); however, there was no effect of drug treatment in the non-reactivated groups (*F*_(1,13)_ = 0.71, *p* = 0.415^c^). This suggests reconsolidation was impaired by systemic MK-801, and by inference that the VR5 reactivation successfully destabilized lever-pressing memory, as there was no significant effect of MK-801 in the absence of reactivation. Orthogonal simple effects showed no significant difference in lever pressing between reactivated and non-reactivated saline- (*F*_(1,13)_ = 1.88, *p* = 0.194^c^) or MK-801- (*F*_(1,14)_ = 2.91, *p* = 0.110^c^) treated animals.

In order to assess general activity during each session, we also performed analysis on nosepoking behavior; any reduction in nosepoking could indicate an impairment in activity or motivation that might have impacted lever-pressing performance independently of any putative instrumental memory reconsolidation deficit. Rats significantly increased their nosepoking during training (*F*_(1,27)_ = 126.28, *p* < 0.001^d^). There was also a significant overall effect of reactivation condition (*F*_(1,27)_ = 4.69, *p* = 0.039^d^) with a significant Treatment × Reactivation interaction (*F*_(1,27)_ = 6.21, *p* = 0.019^d^). There were no other significant group differences during training (Treatment: *F*_(1,27)_ = 1.24, *p*=2.76^d^; Training × Treatment: *F*_(1,27)_ = 2.35, *p* = 0.137^d^; Training × Reactivation: *F*_(1,27)_ = 0.40, *p* = 0.532^d^; Training × Treatment × Reactivation: *F*_(1,27)_ = 0.03, *p* = 0.869^d^). Analysis of the final training day did not reveal any significant effect of drug treatment (*F*_(1,27)_ = 0.20, *p* = 0.655^d^), reactivation condition (*F*_(1,27)_ = 0.74, *p* = 0.396^d^), or interaction between the two (*F*_(1,27)_ = 2.74, *p* = 0.109^d^); therefore, groups displayed similar nosepoking activity on the final day of training, suggesting similar levels of motivation to respond prior to reactivation (data not shown). During the VR5 reactivation, there was no significant acute effect of MK-801 (274.4 ± 39.2) on nosepoking (*F*_(1,14)_ = 0.99, *p* = 0.336^e^) compared to saline controls (223.3 ± 28.7), nor was there any long-term effect of drug treatment on nosepoking activity at test (Fig. 2*B*; Treatment: *F*_(1,27)_ = 1.01, *p* = 0.323^f^; Reactivation: *F*_(1,27)_ = 1.03, *p* = 0.320^f^; Treatment × Reactivation: *F*_(1,27)_ = 0.10, *p* = 0.755^f^), suggesting groups were similarly active during testing.

#### pr-STM

**Figure 3:**
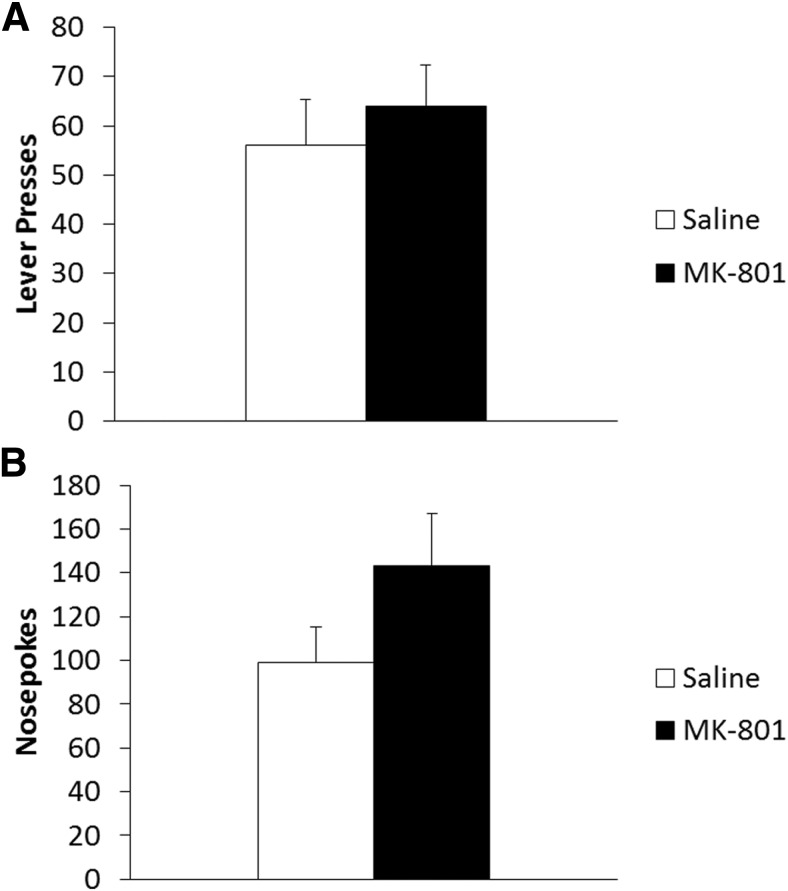
Treatment with systemic MK-801 was without effect on pr-STM. ***A***, Three hours after reactivation MK-801 treated rats (*n* = 7) showed no significant difference in lever pressing compared with vehicle controls (*n* = 9). ***B***, MK-801 had no significant effect on short-term nosepoking behavior 3 h after reactivation. Data are represented as mean number of lever presses + SEM.

An additional group of rats were trained, injected and reactivated as above and their memory tested 3 h after receiving the VR5 reactivation session in order to assess pr-STM. This test controls for any effect of drug treatment on behavioral expression, which may impact later long-term recall. If an impairment in long-term memory is due to disruption of reconsolidation, then short-term memory should be intact. Both treatment groups learned to lever press similarly during training (data not shown; Training: *F*_(1,14)_ = 461.37, *p* < 0.001^g^; Treatment: *F*_(1,14)_ = 1.08, *p* = 0.317^g^; Training × Treatment: *F*_(1,14)_ = 0.58, *p* = 0.461^g^). There was no significant effect of MK-801 on lever-pressing performance during either the VR5 reactivation (Saline: 91.2 ± 4.5; MK-801: 100.9 ± 4.5; *F*_(1,14)_ = 2.24, *p* = 0.157^h^) or pr-STM test (Fig. 3*A*; *F*_(1,14)_ = 0.38, *p* = 0.548^i^). Thus, pr-STM was unimpaired by MK-801 treatment prior to the VR5 reactivation.

Similar analysis was performed for nosepoking to confirm MK-801 had no significant effect on short-term behavioral expression. Nosepoking activity was similar in both treatment groups during training (data not shown; Training: *F*_(1,14)_ = 9.47, *p* = 0.008^j^; Treatment: *F*_(1,14)_ = 0.001, *p* = 0.974^j^; Training × Treatment: *F*_(1,14)_ = 0.01, *p* = 0.927^j^). No acute effect of MK-801 was observed in nosepoking activity during the VR5 reactivation (Saline: 245.8 ± 19.6; MK-801: 238.3 ± 20.2; *F*_(1,14)_ = 0.07, *p* = 0.797^k^), nor in the test of pr-STM (Fig. 3*B*; *F*_(1,14)_ = 2.47, *p* = 0.139^l^).

#### Non-reinforced reactivation

Following the success of the VR5 reactivation to destabilize the briefly-trained lever-pressing memory, we next sought to test for the necessity of reinforcer presentation during reactivation. In many cases, pavlovian memories have been successfully destabilized and their reconsolidation disrupted using non-reinforced stimulus presentation (Lee et al., 2006b; Milton et al., 2008b). A recent study found that non-reinforced reactivation did not destabilize a well-learned instrumental memory (Exton-McGuinness et al., 2014b). However, it remains possible that shorter non-reinforced sessions could destabilize instrumental memories. Session length plays an important part in determining the switch between destabilization, leading to memory updating, and extinction (Flavell and Lee, 2013; Merlo et al., 2014), in which a new inhibitory memory suppresses behavioral responding (Bouton, 2002).

**Figure 4:**
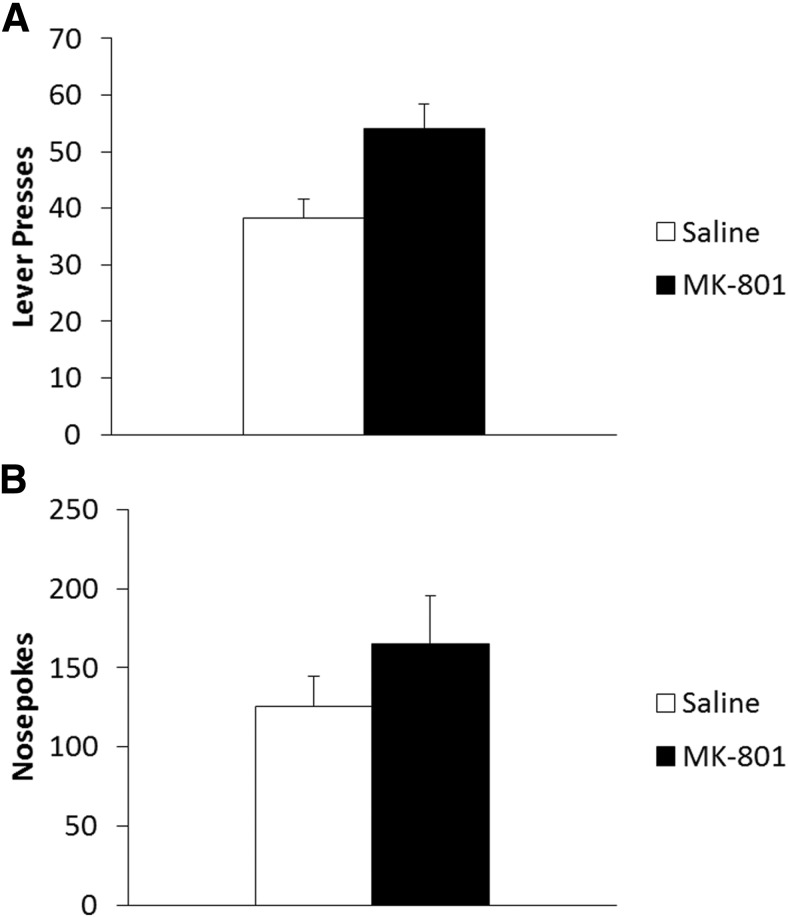
MK-801 impaired new extinction learning resulting from the non-reinforced reactivation. ***A***, MK-801-injected rats (*n* = 6) responded significantly more than saline controls at test (*n* = 8). ***B***, MK-801 treatment had no significant effect on nosepoking at test. Data are represented as mean number of lever presses + SEM.

During the training phase, repeated-measures ANOVA revealed a significant effect of Training (*F*_(1,12)_ = 698.0, *p* < 0.001^m^), showing rats learned the lever-pressing task. A Training × Treatment interaction (*F*_(1,12)_ = 5.04, *p* = 0.044^m^) was revealed with no main effect of Treatment (*F*_(1,12)_ = 0.06, *p* = 0.815^m^). Analysis of the second day of training showed no significant difference in lever pressing (*F*_(1,12)_ = 1.49, *p* = 0.245^m^), indicating groups were similarly performing at the end of training prior to reactivation the next day (data not shown). ANOVA of the reactivation session revealed a significant increase in lever pressing following MK-801 injection (Saline: 5.1 ± 1.7; MK-801: 16.3 ± 2.6; *F*_(1,12)_ = 14.19, *p* = 0.003^n^), which persisted in the test session 24 h later (Fig. 4*A*; *F*_(1,12)_ = 8.23, *p* = 0.014^°^).

Accompanying analysis of nosepoking showed rats significantly increased their nosepoking over Training (*F*_(1,12)_ = 31.45, *p* < 0.001^p^) with no significant group differences (data not shown; Treatment: *F*_(1,12)_ = 2.62, *p* = 0.132^p^; Training × Treatment: *F*_(1,12)_ = 0.09, *p* = 0.765^p^), implying similar activity levels prior to drug intervention and reactivation. During reactivation, MK-801-injected rats nosepoked significantly more than saline controls (Saline: 20.8 ± 4.8; MK-801: 36.5 ± 3.0; *F*_(1,12)_ = 6.44, *p* = 0.026^q^); however, this effect had dissipated by the test session (Fig. 4*B*; *F*_(1,12)_ = 1.37, *p* = 0.264^r^).

#### FR1 reactivation

Given that the non-reinforced reactivation did not appear to destabilize lever-pressing memory, allowing its reconsolidation to be disrupted by MK-801, we next tested whether the memory could be destabilized by a brief FR1 reactivation. In the case of memories that are not well-learned, training trials have been used to destabilize memory traces (Rodriguez-Ortiz et al., 2005; Exton-McGuinness et al., 2014a). This session was fundamentally equivalent to a short training session and also tested for the sufficiency of reinforcer presentation in the destabilization of lever-pressing memory.

**Figure 5:**
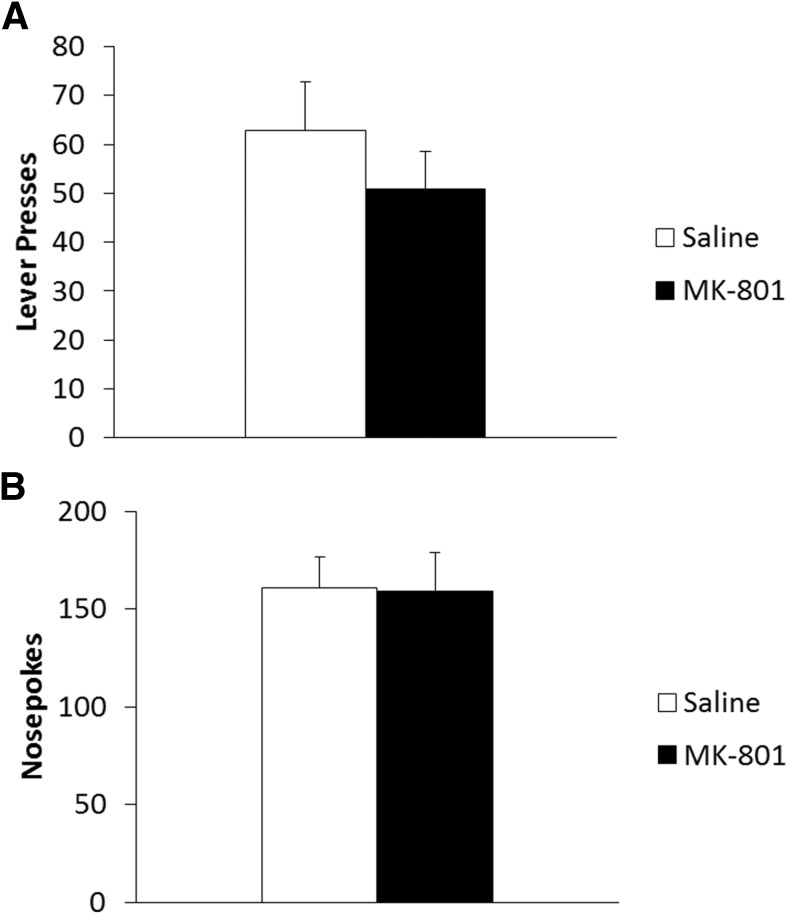
MK-801 was without effect when administered prior to a brief FR1 reactivation. ***A***, MK-801 (*n* = 6) and saline-treated (*n* = 6) groups showed no significant difference in performance at test. ***B***, MK-801 administration was without effect on nosepoking at test. Data are represented as mean + SEM.

Both treatment groups acquired similar levels of lever pressing during training (data not shown; Training: *F*_(1,10)_ = 341.8, *p* < 0.001^s^; Treatment: *F*_(1,10)_ = 0.54, *p* = 0.481^s^; Training × Treatment: *F*_(1,10)_ = 0.57, *p* = 0.468^s^). Rats were then injected with MK-801 or saline, followed by the FR1 reactivation session. During reactivation, all rats made the maximum of 20 lever presses and acquired 20 sucrose pellets each. The next day at test (Fig. 5*A*), there was no significant difference in lever pressing between treatment groups (*F*_(1,10)_ = 0.90, *p* = 0.365^t^), implying the FR1 session did not destabilize the lever-pressing memory, preventing MK-801 from disrupting its reconsolidation.

Companion analysis showed nosepoking to increase through training (*F*_(1,10)_ = 41.53, *p* < 0.001^u^), with no significant difference between groups (data not shown; Treatment: *F*_(1,10)_ = 0.46, *p* = 0.514^u^; Training × Treatment: *F*_(1,10)_ = 1.18, *p* = 0.304^u^), nor were there any significant group differences during reactivation (Saline: 84.7 ± 9.5; MK-801: 119.3 ± 14.7; *F*_(1,10)_ = 3.92, *p* = 0.076^v^) or the test session (Fig. 5*B*; *F*_(1,10)_ = 0.004, *p* = 0.949^w^).

### Intra-accumbans study

#### Drug infusions

Having established the ability of the VR5 reactivation to destabilize the weakly-trained lever-pressing memory, we next investigated the potential local involvement of accumbal NMDA and D1Rs in reconsolidation of lever-pressing memory. Previous work has shown coactivation of NMDA and D1Rs in the NAc to be involved in the acquisition of lever pressing (Smith-Roe and Kelley, 2000), and we sought to investigate whether such coactivation also played a role in reconsolidation by infusing AP-5, SCH23390, and a combination of the two into the NAc immediately prior to the VR5 reactivation. We also infused MK-801 alone directly into the NAc in order to test whether the NAc was a central locus of action for systemic MK-801.

There were no significant differences in lever pressing between the infusion groups during training (data not shown; Training: *F*_(1,24)_ = 711.3, *p* < 0.001^x^; Treatment: *F*_(4,24)_ = 0.96, *p* = 0.447^x^; Training × Treatment: *F*_(4,24)_ = 0.78, *p* = 0.550^x^). As there was a single common PBS vehicle control, we conducted Bonferroni-corrected planned comparisons between the vehicle group and each drug group (effective *p* < 0.0125). No treatment group significantly differed in lever pressing from the PBS control on the final day of training: MK-801 (*F*_(1,10)_ = 0.16, *p* = 0.700^x^), AP-5 (*F*_(1,9)_ = 0.82, *p* = 0.389^x^), SCH23390 (*F*_(1,10)_ = 0.03, *p* = 0.860^x^), AP-5/SCH23390 (*F*_(1,10)_ = 1.13, *p* = 0.314^x^). During the VR5 reactivation session, an overall ANOVA revealed a significant effect of drug treatment (*F*_(4,24)_ = 10.69, *p* < 0.001^y^). Planned comparisons showed that co-infusion of AP-5 and SCH23390 immediately prior to reactivation acutely reduced lever pressing during the VR5 session (11.3 ± 5.9; *F*_(1,10)_ = 27.7, *p* < 0.001^y^); however, infusions of MK-801 (82.0 ± 8.4; *F*_(1,10)_ = 0.13, *p* = 0.725^y^), AP-5 (66.6 ± 10.6; *F*_(1,9)_ = 0.45, *p* = 0.519^y^) or SCH23390 (69.8 ± 8.1; *F*_(1,10)_ = 0.28, *p* = 0.611^y^) alone had no acute effect compared to PBS-infused controls (77.0 ± 11.0).

**Figure 6:**
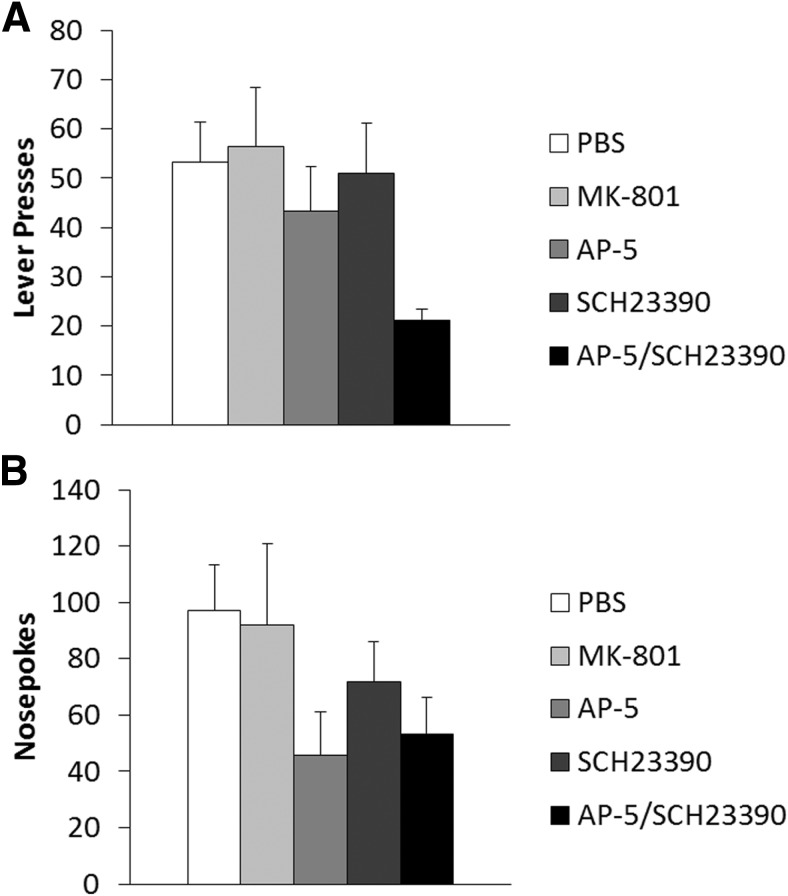
Combined infusion of AP-5/SCH23390 significantly impaired behavioral activity when administered immediately prior to the VR5 reactivation. ***A***, Coinfusion of AP-5/SCH23390 (*n* = 6) significantly impaired lever pressing at test compared with PBS controls (*n* = 6). Infusions of MK-801 (*n* = 6), AP-5 (*n* = 5), or SCH23390 (*n* = 6) alone were without significant long-term effect on lever pressing. ***B***, There was no significant evidence for any long-term impairment in nosepoking with any infusion. Data are represented as mean + SEM.

At test, 24 h after reactivation, although an overall ANOVA did not provide conclusive evidence for a long-term effect of infusion (*F*_(4,24)_ = 2.60, *p* = 0.06^z^), planned comparisons revealed a significant reduction in lever pressing in rats previously infused with the AP-5/SCH23390 in combination compared to PBS vehicle controls (Fig. 6*A*; *F*_(1,10)_ = 14.1, *p* = 0.004^z^). In contrast, similar planned comparisons did not show any lever-pressing impairment at test in rats given infusions of MK-801 (*F*_(1,10)_ = 0.05, *p* = 0.823^z^), AP-5 (*F*_(1,9)_ = 0.65, *p* = 0.441^z^) or SCH23390 (*F*_(1,10)_ = 0.03, *p* = 0.871^z^) alone (Fig. 6*A*).

Mirror analysis of nosepoking responses showed similar activity across infusion groups during training, suggesting all groups were similarly motivated prior to reactivation (data not shown; Training: *F*_(1,24)_ = 42.00, *p* < 0.001^aa^; Treatment: *F*_(4,24)_ = 1.46, *p* = 0.245^aa^; Training × Treatment: *F*_(4,24)_ = 0.259, *p* = 0.901^aa^). Planned comparisons on the final day of training did not reveal any significant differences in nosepoking of any treatment group compared to PBS controls (MK-801: *F*_(1,10)_ = 0.09, *p* = 0.771^aa^; AP-5: *F*_(1,9)_ = 2.36, *p* = 0.159^aa^; SCH23390: *F*_(1,10)_ = 0.11, *p* = 0.746^aa^; AP-5/SCH23390: *F*_(1,10)_ = 2.23, *p* = 0.166^aa^).

Overall ANOVA of nosepoking during the VR5 reactivation revealed a significant acute effect of infusion (*F*_(4,24)_ = 8.14, *p* < 0.001^bb^). Planned comparisons revealed co-infusion of AP-5/SCH23390 acutely impaired nosepoking at reactivation (48.8 ± 16.7; *F*_(1,10)_ = 23.68, *p* < 0.001^bb^), but infusion of MK-801 (284.3 ± 47.3; *F*_(1,10)_ = 1.02, *p* = 0.335^bb^), AP-5 (161.4 ± 22.2; *F*_(1,9)_ = 2.51, *p* = 0.147^bb^) or SCH23390 (203.7 ± 25.8; *F*_(1,10)_ = 0.30, *p* = 0.596^bb^) alone had no acute effect compared to PBS controls (213.6 ± 36.4). This would imply that the rats co-infused with AP-5/SCH23390 were generally less active during the reactivation session, which may also have affected their rate of lever pressing.

On the test session, overall ANOVA of nosepoking did not reveal any long-term effect of prior infusion (Fig. 6*B*; *F*_(4,24)_ = 1.42, *p* = 0.257^cc^). Planned comparisons did not show any significant effect of any drug on nosepoking during the test session (MK-801: *F*_(1,10)_ = 0.03, *p* = 0.875^cc^; AP-5: *F*_(1,9)_ = 5.198, *p* = 0.049^cc^; SCH23390: *F*_(1,10)_ = 0.13, *p* = 0.728^cc^; AP-5/SCH23390: *F*_(1,10)_ = 4.51, *p* = 0.060^cc^).

#### Non-reactivation control

In order to test the reactivation dependence of the combined AP-5/SCH23390 infusion on lever pressing, an additional group of rats were trained and given an infusion of PBS or AP-5/SCH23390 in the absence of any behavioral session. Results were compared to the reactivated PBS and AP-5/SCH23390 groups from the previous experiment. If the effect of intra-NAc AP-5/SCH23390 was to impair reconsolidation, then there should be no effect of the infusion in the absence of behavioral reactivation (memory destabilization).

Both reactivated and non-reactivated groups increased their lever pressing during Training (*F*_(1,20)_ = 950.7, *p* < 0.001^dd^). There were no significant main effects of Treatment (*F*_(1,20)_ = 1.93, *p* = 0.180^dd^) or Reactivation (*F*_(1,20)_ = 0.20, *p* = 0.659^dd^) on lever pressing during training, nor were there any Treatment × Reactivation (*F*_(1,20)_ = 0.178, *p* = 0.678^dd^), Training × Treatment (*F*_(1,20)_ = 0.05, *p* < 0.824^dd^) or Training × Treatment × Reactivation (*F*_(1,20)_ = 1.11, *p* = 0.305^dd^) interactions; however, there was a significant Training × Reactivation interaction (*F*_(1,20)_ = 8.56, *p* = 0.008^dd^). Analysis of lever pressing on the second day of training showed no significant differences between reactivated and non-reactivated infusion groups (data not shown; Treatment: *F*_(1,20)_ = 1.40, *p* = 0.250^dd^; Reactivation: *F*_(1,20)_ = 3.96, *p* = 0.060^dd^; Treatment × Reactivation: *F*_(1,20)_ = 0.84, *p* = 0.371^dd^), indicating experimental groups were at similar levels of performance prior to drug infusion.

**Figure 7:**
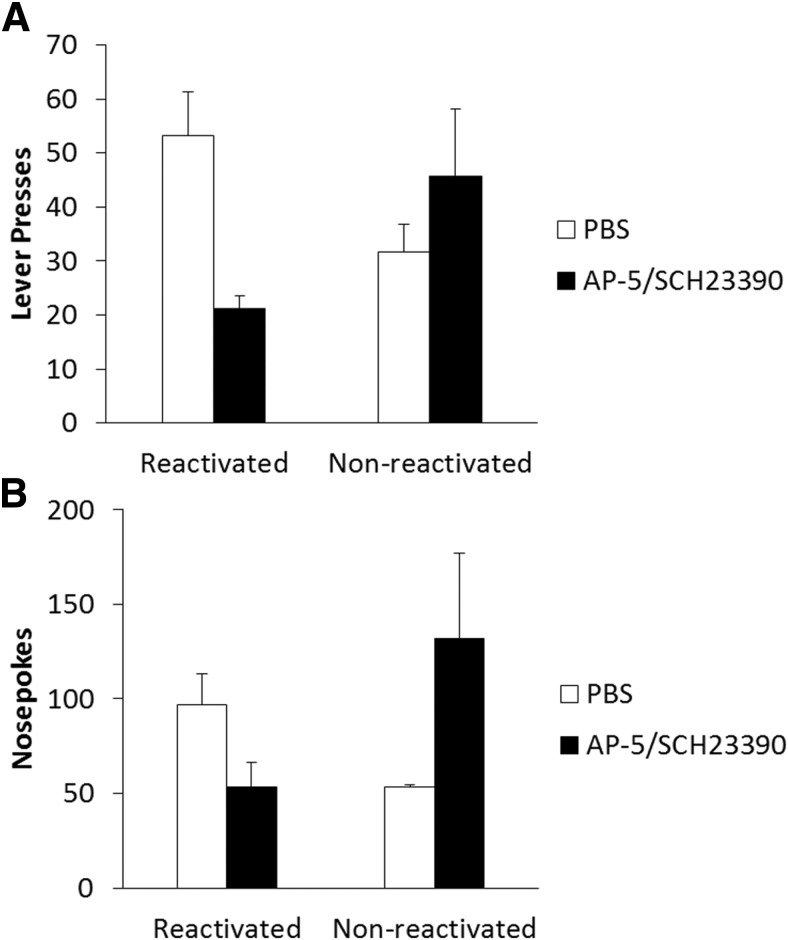
The effect of combined AP-5/SCH23390 infusion to impair responding was critically dependent on memory reactivation. ***A***, The combined infusion significantly impaired lever pressing at test (as shown in Fig. 6, presented again here for clarity); however, coadministration of AP-5/SCH23390 (*n* = 7) did not significantly impair lever pressing if given in the absence of memory reactivation, compared with non-reactivated PBS infused controls (*n* = 5). ***B***, Combined infusion of AP-5/SCH23390 also had a reactivation-dependent effect on nosepoking. Nosepoking was moderately impaired in the reactivated infusion group; however, the reactivation dependence of the effect is primarily driven by low responding in the non-reactivated vehicle group. Data are represented as mean + SEM.

Combined analysis of lever pressing at test, for both reactivated and non-reactivated groups, revealed a reactivation-dependent effect of AP-5/SCH23390 infusion (Fig. 7*A*; Treatment × Reactivation: *F*_(1,20)_ = 6.82, *p* = 0.017^ee^), with no main effects of Treatment (*F*_(1,20)_ = 1.03, *p* = 0.323^ee^) or Reactivation (*F*_(1,20)_ = 0.03, *p* = 0.868^ee^). Analysis of simple effects showed significantly reduced lever pressing in rats infused with AP-5/SCH23390 immediately prior to the VR5 reactivation compared with their PBS-infused controls (*F*_(1,10)_ = 14.1, *p* = 0.004^ee^); importantly, there was no significant effect of AP-5/SCH23390 infusion on lever pressing in non-reactivated rats (*F*_(1,10)_ = 0.83, *p* = 0.385^ee^), suggesting reconsolidation was disrupted by the infusion. Orthogonal simple effects showed no significant difference between reactivated and non-reactivated groups given either PBS (*F*_(1,9)_ = 4.44, p=0.064^ee^) or AP-5/SCH23390 (*F*_(1,11)_ = 3.22, *p* = 0.100^ee^).

Similar combined analysis of nosepoking for reactivated and non-reactivated groups showed no significant differences during training (data not shown; Training: *F*_(1,20)_ = 40.44, *p* < 0.001^ff^; Treatment: *F*_(1,20)_ = 3.33, *p* = 0.083^ff^; Reactivation: *F*_(1,20)_ = 1.59, *p* = 0.222^ff^; Training × Treatment: *F*_(1,20)_ = 1.26, *p* = 0.275^ff^; Training × Reactivation: *F*_(1,20)_ = 0.05, *p* = 0.833^ff^; Training × Treatment × Reactivation: *F*_(1,20)_ = 1.06, *p* = 0.315^ff^), suggesting all groups were similarly motivated prior to infusion.

Combined analysis of nosepoking at test, 24 h after infusion, (Fig. 7*B*) revealed a significant reactivation-dependent effect of AP-5/SCH23390 (Treatment × Reactivation: *F*_(1,20)_ = 4.71, *p* = 0.042^gg^), with no main effects of Treatment (*F*_(1,20)_ = 0.41, *p* = 0.531^gg^) or Reactivation (*F*_(1,20)_ = 0.34, *p* = 0.567^gg^). Analysis of simple effects did not reveal any significant differences in nosepoking between AP-5/SCH23390 and PBS-infused reactivated groups (*F*_(1,10)_ = 4.51, *p* = 0.060^gg^), nor in non-reactivated controls (*F*_(1,10)_ = 2.24, *p* = 0.165^gg^). Analysis of orthogonal simple effects revealed significantly reduced nosepoking in non-reactivated PBS-infused rats compared with their reactivated counterparts (*F*_(1,9)_ = 6.58, *p* = 0.030^gg^). This comparison was not significant for rats given the combined AP-5/SCH23390 infusion (*F*_(1,11)_ = 2.47, *p* = 0.144^gg^). The effect on AP-5/SCH23390 infusion on nosepoking suggests the infusion did result in impaired motivation in some groups; however, given the reactivation-dependence of the effect, it may have been mediated by a reconsolidation mechanism.

### Cocaine study

Using our findings from the sucrose setting, we sought to expand our research to investigate whether a similar VR5 reactivation could be used to destabilize weakly-learned lever-pressing memory for cocaine self-administration, such that systemic MK-801 might impair the reconsolidation of the instrumental cocaine memory and lead to a long-term reduction in cocaine seeking.

During training, both treatment groups learned to lever press for cocaine at similar rates with no significant differences in performance prior to reactivation (data not shown; Training: *F*_(1,22)_ = 11.23, *p* = 0.003^hh^; Treatment: *F*_(1,22)_ = 0.14, *p* = 0.708^hh^; Reactivation: *F*_(1,22)_ = 0.46, *p* = 0.504^hh^; Treatment × Reactivation: *F*_(1,22)_ = 0.02, *p* = 0.905^hh^; Training × Treatment: *F*_(1,22)_ = 1.85, *p* = 0.188^hh^; Training × Reactivation: *F*_(1,22)_ = 0.83, *p* = 0.374^hh^; Training × Treatment × Reactivation: *F*_(1,22)_ = 0.04, *p* = 0.842^hh^). The day after the final training session, rats were injected with MK-801 or saline vehicle 30 min prior to reactivation. During the VR5 reactivation, MK-801- (26.9 ± 2.3) and saline- (24.3 ± 2.3) treated rats displayed similar lever-pressing performance (*F*_(1,12)_ = 0.63, *p* = 0.444^ii^).

**Figure 8:**
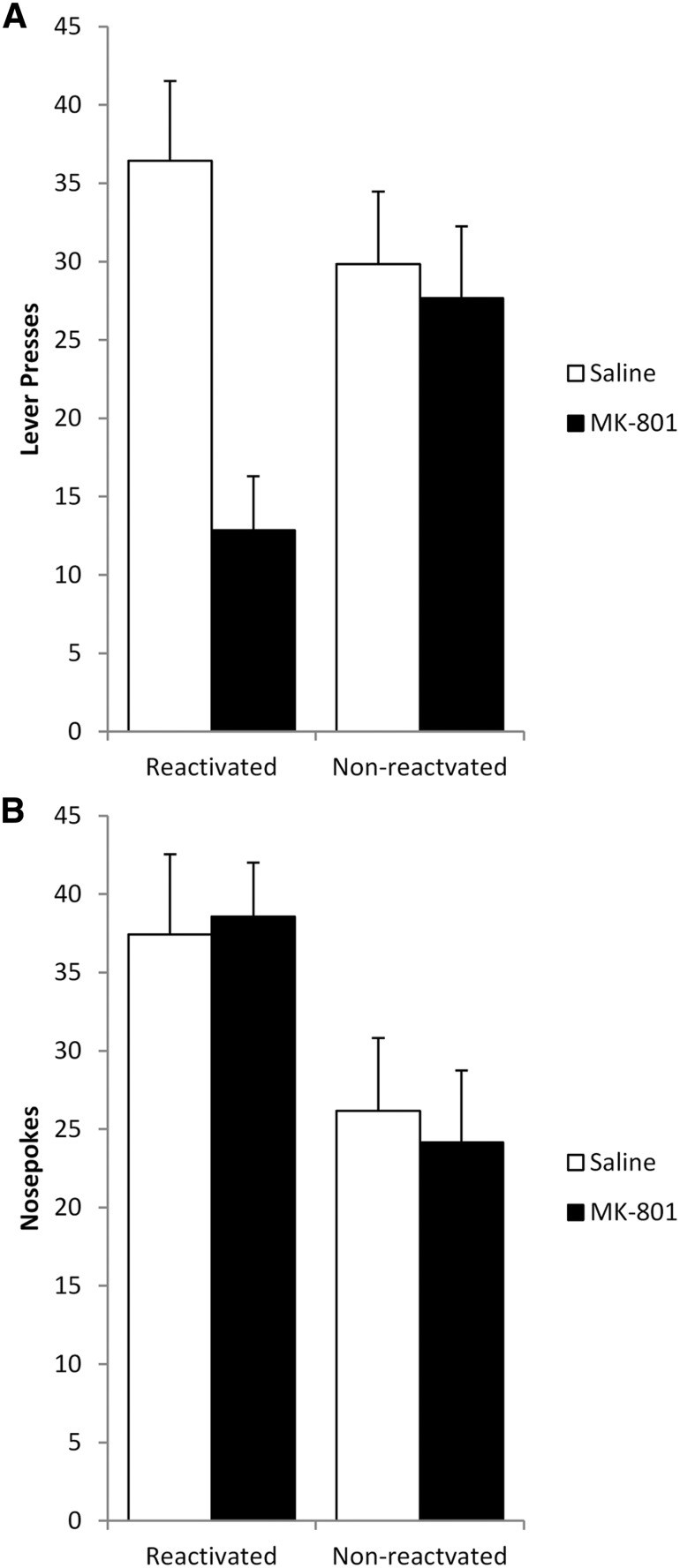
Administration of MK-801, prior to a shift to a VR5 schedule of reinforcement, significantly impaired the reconsolidation of long-term lever pressing for cocaine self-administration in a reactivation-dependent manner. ***A***, Lever pressing at test was significantly reduced in rats administered MK-801 prior to the VR5 reactivation (*n* = 7) compared with both reactivated vehicle-injected rats (*n* = 7) and the non-reactivated MK-801-treated group (*n* = 6). Non-reactivated vehicle rats (*n* = 6) showed similar performance to their reactivated counterparts. ***B***, Treatment with MK-801 had no significant effect on nosepoking behavior regardless of when animals received memory reactivation. Data are represented as mean + SEM.

At test 24 h later (Fig. 8*A*), ANOVA of lever pressing revealed a significant Treatment × Reactivation interaction (*F*_(1,22)_ = 6.70, *p* = 0.017^jj^) with a main effect of MK-801 Treatment (*F*_(1,22)_ = 9.69, *p* = 0.005^jj^), but no main effect of Reactivation (*F*_(1,22)_ = 0.99, *p* = 0.331^jj^). Analysis of simple effects showed lever pressing to be significantly reduced in rats given MK-801 prior to the VR5 reactivation (Fig. 8*A*; *F*_(1,12)_ = 17.10, *p* = 0.001^jj^); however, MK-801 had no effect in the absence of reactivation (*F*_(1,10)_ = 0.132, *p* = 0.724^jj^), indicative of a reconsolidation impairment. Orthogonal simple effects showed the lever pressing of reactivated MK-801-treated rats to be significantly lower than their non-reactivated counterparts (*F*_(1,11)_ = 8.19, *p* = 0.015^jj^); however, saline-treated animals showed similar lever pressing performance regardless of reactivation condition (*F*_(1,11)_ = 1.05, *p* = 0.328^jj^).

Companion analysis of nosepoking behavior was performed to assess general activity during each session. During training, ANOVA revealed a significant main effect of Reactivation (*F*_(1,22)_ = 4.64, *p* = 0.042^kk^); however, there were no other significant differences between experimental groups (Training: *F*_(1,22)_ = 2.80, *p* = 0.109^kk^; Treatment: *F*_(1,22)_ = 0.40, *p* = 0.533^kk^; Training × Treatment: *F*_(1,22)_ = 0.40, *p* = 0.535^kk^; Training × Reactivation: *F*_(1,22)_ = 3.09, *p* = 0.093^kk^; Treatment × Reactivation: *F*_(1,22)_ = 0.27, *p* = 0.612^kk^; Training × Treatment × Reactivation: *F*_(1,22)_ = 0.05, *p* = 0.829^kk^). Analysis of the second training day showed reactivated groups nosepoked significantly more than non-reactivated controls (*F*_(1,22)_ = 4.63, *p* = 0.043^kk^); however, there was no significant difference in the number of nosepokes between drug groups (Treatment: *F*_(1,22)_ = 0.47, *p* = 0.499^kk^; Treatment × Reactivation: *F*_(1,22)_ = 0.18, *p* = 0.680^kk^). This shows non-reactivated groups were generally less active than their reactivated counterparts prior to injection; however, it was not specific to one treatment group.

Reactivated saline- (30.6 ± 11.7) and MK-801- (51.0 ± 13.0) injected rats showed similar nosepoking performance during the VR5 reactivation (*F*_(1,12)_ = 1.36, *p* = 0.266^ll^). Finally, nosepoking performance during the test session was similar in all experimental groups (Fig. 8*B*; Treatment: *F*_(1,22)_ = 0.004, *p* = 0.951^mm^; Reactivation: *F*_(1,22)_ = 3.42, *p* = 0.078^mm^; Treatment × Reactivation: *F*_(1,22)_ = 0.05, *p* = 0.823^mm^), indicating there were no significant differences in general activity during the test session. Thus, the specific effect on lever pressing likely represents a reduction in cocaine seeking, rather than an overall reduction in motor activation.

## Discussion

The present results demonstrate that the reconsolidation of a weakly-trained lever-pressing memory for sucrose reinforcement can be disrupted by systemic, but not intra-NAc, MK-801. By inference, the shift to a VR5 schedule was sufficient to cause the memory to destabilize, rendering it labile and susceptible to the amnestic effect of MK-801. Notably, the amnestic effect of MK-801 was reactivation-dependent, indicating the amnestic effect was due to an impairment of reconsolidation and was specific to lever pressing with no significant effect on nosepoking responses. A disruption of lever pressing for sucrose was also observed following pre-reactivation intra-NAc coinfusion of AP-5/SCH23390, suggesting a possible role for coactivation of accumbal D1Rs and NMDARs in the reconsolidation process. The VR5 reactivation also proved effective in destabilizing memory for cocaine self-administration, as demonstrated by a systemic MK-801-induced reconsolidation impairment. This provides an important demonstration for the viability of reconsolidation-based therapies for maladaptive seeking behaviors that target the instrumental components of memory.

Our results provide evidence that weakly-trained lever-pressing memories undergo reactivation-induced destabilization and subsequent reconsolidation under similar conditions to well-trained instrumental memories (Exton-McGuinness et al., 2014b). The reconsolidation of lever pressing is shown here to be impaired by systemic MK-801 and intra-NAc AP-5/SCH23390 administered shortly before VR5 reactivation. The amnestic effect of these treatments was critically dependent on memory reactivation, a key criterion for assessing reconsolidation deficits (Dudai, 2004); treatment under inappropriate reactivation parameters or in the absence of reactivation produced no subsequent impairment in responding. The lack of effect in non-reactivated controls also demonstrates that the behavioral impairments at test were not due to any non-specific effects of drug administration. There was a general acute effect of systemic MK-801 to moderately elevate responding during the reactivation sessions, significantly so with the non-reinforced reactivation. However, this is likely explained by the hyperactivity caused at doses used in our experiment (Hargreaves and Cain, 1995). Importantly, any acute arousing effect of MK-801 is short-lived, as confirmed by the absence of any significant effect at pr-STM, and cannot account for differences observed 24 h later at test.

While the VR5 session might be expected to enhance responding via additional learning, as lever presses were reinforced (although at a reduced rate compared to training), there was only weak evidence for this and visual increases in the performance of saline controls were not statistically significant. Reactivated vehicle-treated groups did respond moderately above the level of non-reactivated controls for both sucrose and cocaine reinforced memories, suggesting some degree of additional learning, whether by a reconsolidation-mediated process or other mechanism. However, it cannot be interpreted that the amnestic effect of MK-801 was due solely to a disruption of additional learning. First, non-reactivated drug-treated groups (with no opportunity for any additional learning) responded at a similar level to reactivated vehicle groups. Second, the deficits observed at test in reactivated treatment-impaired groups appear to be driven, at least in part, by a reduction in responding compared with non-reactivated animals, consistent with a canonical reconsolidation impairment. Furthermore, in the cocaine setting, differences in nosepoking during training between reactivated and non-reactivated groups may indicate differences in pre-reactivation motivation, which may have contributed to the apparent visual increase in lever pressing in the reactivated vehicle group at test. Finally, pr-STM following the VR5 reactivation in the sucrose-seeking setting showed no evidence for acquisition of additional learning, nor any impairment of performance. Intact pr-STM supports the conclusion that it was the reconsolidation of long-term memory that was disrupted by MK-801; any impairment of additional learning during reactivation would also be expected to be observed at this time point, given that MK-801 impairs the acquisition of new memories (Gould et al., 2002; Mackes and Willner, 2006; Alaghband and Marshall, 2013).

The reactivation-dependent nature of the lever-pressing deficit confirms the effect of treatment was to impair reconsolidation; however, it is not immediately clear whether pavlovian or instrumental memory was impaired. While instrumental memories encode associations between the behavioral response and reward, for example lever pressing and cocaine, pavlovian associations store information about salient environmental stimuli or contexts. These pavlovian memories mediate a variety of behavioral effects, including orientating or approach to a conditioned stimulus (Cleland and Davey, 1983), such as a lever. Indeed, pavlovian associations are capable of supporting the act of lever pressing when the lever, or some aspect of the lever, acts as a conditioned stimulus (Davey et al., 1981). Pavlovian learning can also impact motivation and activity. While this can function to generally increase (or decrease) overall activity, it can also act to modulate the vigor of specific instrumental behaviors associated with a specific outcome (Dickinson and Balleine, 1994; Corbit et al., 2007). Consequently, while we might intuitively interpret reductions in lever pressing as reconsolidation deficits in the underlying instrumental association (Exton-McGuinness et al., 2014b), they could equally have been mediated via disruption of pavlovian memories that modulate instrumental behaviors; importantly, these pavlovian memories are known to undergo reconsolidation (Lee and Everitt, 2008b; Fuchs et al., 2009; Milton et al., 2012). In our study, nosepoke responses provide an indirect measure of pavlovian memory strength through measuring general activity (and in the case of the sucrose studies, approach to the reward location). Were both nosepokes and lever presses impaired in a reactivation-dependent manner, then it would be highly likely that it was the reconsolidation of the pavlovian, rather than instrumental, components of lever pressing memory that was disrupted.

In the case of the systemic studies, using both sucrose and cocaine reinforcement, the reactivation-dependent effect was specific to lever pressing, with no significant difference in nosepoking between groups. As the reconsolidation impairment manifests only in lever pressing, it is likely it was the instrumental memory that was disrupted in these experiments. The selectivity of the MK-801 effect only with the VR5 reactivation is particularly important in this respect, given that MK-801 impairs pavlovian memory reconsolidation when reactivation consists of re-exposure to relevant stimuli and contextual cues (Lee and Everitt, 2008b, 2008c), with (Exton-McGuinness et al., 2014a) or without (Reichelt and Lee, 2012) concomitant sucrose presentation. Moreover, one would have expected pavlovian memories to have been destabilized by the non-reinforced and FR1 reactivation conditions. However this does not appear to have been the case, supporting the conclusion both that the non-reinforced and FR1 reactivations were insufficient to cause lever-pressing memory to destabilize, and that the impairment with the VR5 reactivation was in instrumental memory.

In the non-reinforced reactivation condition, MK-801-treated rats responded significantly more than vehicle controls at test. This increase may be explained as an impairment of extinction learning by MK-801, rather than reconsolidation. NMDAR antagonists have previously been shown to impair extinction (Lissek and Güntürkün, 2003; Kelamangalath et al., 2007) leading to elevated responding at test. While brief non-reinforced sessions are conventionally used to destabilize pavlovian memories, the fact that saline-treated animals given the non-reinforced reactivation showed low responding compared to both the FR1 and VR5 conditions suggests they did extinguish during the non-reinforced reactivation, despite its brevity. While it may be surprising that such a brief session was sufficient to cause extinction learning, the lever-pressing response was only weakly trained in our experiment and thus likely easily extinguished. Given that extinction and reconsolidation may be competing processes (Eisenberg et al., 2003; Pedreira and Maldonado, 2003), both of which are impaired by MK-801 in appetitive settings (Flavell and Lee, 2013), it seems unlikely that the non-reinforced reactivation destabilized memory. However, it remains possible that shorter non-reinforced sessions that do not result in extinction learning could destabilize the instrumental memory.

The FR1 reactivation also did not destabilize the instrumental trace. This is important, as the destabilization resulting from the VR5 reactivation cannot simply be attributed to the presence of the reinforcer. Training trials have been shown to destabilize fear memories (Duvarci and Nader, 2004; Eisenberg and Dudai, 2004; Lee, 2008), appetitive pavlovian (Rodriguez-Ortiz et al., 2005; Milekic et al., 2006; Valjent et al., 2006), and object recognition memories (Kelly et al., 2003; Akirav and Maroun, 2006). However, full-length (Hernandez and Kelley, 2004) and brief (Mierzejewski et al., 2009) training sessions have proved insufficient in instrumental settings, consistent with our findings in this study. The lack of effect with the FR1 reactivation suggests that the salient feature of the VR5 reactivation, contributing to its ability to destabilize instrumental memory, was the unpredictability within the schedule. Recent studies have demonstrated that a change in the predictability of the unconditioned stimulus appears to be associated with increased chances of pavlovian memory destabilization (Díaz-Mataix et al., 2013; Sevenster et al., 2013); this may contribute to the generation of a prediction error, believed to be required for memories to destabilize (Exton-McGuinness et al., 2014a). Alternatively, the change in contingency could provide new information that may be required for initiation of memory reconsolidation (Pedreira et al., 2004; Lee, 2009; Winters et al., 2009).

In the case of the intra-accumbal study, the precise nature of the impairment is less clear, as there was a reactivation-dependent effect of AP-5/SCH23390 infusion on both lever pressing and nosepoking. While the effect on nosepoking appears to be mostly driven by a reduction in the non-reactivated PBS group, there does appear to be a moderate, although inconclusive (*p* = 0.06), reduction of nosepoking in the reactivated AP-5/SCH23390 group. Moreover, intra-accumbal MK-801 had no significant long-term effect on behavior, suggesting both that the NAc was not a central locus of action for systemic MK-801 and that the effect of intra-NAc AP-5/SCH23390 was mediated by a different mechanism than that of systemic MK-801. While the long-term effect of AP-5/SCH23390 on lever pressing appears more severe than its effect on nosepoking, it seems likely that the effect on lever pressing was driven, at least in part, by a reduction in vigor resulting from the impairment of one or more pavlovian components of behavior. The NAc is strongly implicated in motivation and arousal (Balleine and Killcross, 1994; Cardinal et al., 2003), and the pavlovian interpretation is further supported by the strong acute effect of AP-5/SCH23390 at reactivation, impairing again both lever pressing and nosepoking. The apparent persistence of the impairment from reactivation to test does not seem to be due to damage to the NAc, however, as the effect on both lever pressing and nosepoking was reactivation-dependent, implying the deficit did result from a reconsolidation impairment in pavlovian memory.

If the effect of accumbal AP-5/SCH23390 was to disrupt pavlovian memory reconsolidation, this raises two questions. First, did the VR5 reactivation destabilize both pavlovian and instrumental components of memory simultaneously, and did it do so in all experimental studies? Second, why did AP-5 and SCH23390 given alone not also impair the reconsolidation of the pavlovian memory, given that these have previously been shown to disrupt the consolidation of appetitive memory when infused into the NAc (Kelley et al., 1997; Smith-Roe and Kelley, 2000; Dalley et al., 2005)? To the first question, it seems unlikely that both pavlovian and instrumental associations were destabilized simultaneously, given the dose of MK-801 in the systemic studies is well established to impair reconsolidation of pavlovian memories (Lee et al., 2006b; Milton et al., 2008a) and there was no evidence of a pavlovian memory impairment in the systemic studies. It should be noted, however, that the question of the capacity of a single treatment to disrupt the reconsolidation of more than one memory representation has not been adequately addressed in the literature. Nevertheless, it is perhaps most parsimonious to conclude that the VR5 reactivation destabilized only the instrumental component of behavior.

In attempting to address the second question, it is important to note that our drugs were delivered prior to reactivation. This is a key limitation to our experimental design, as treatments may impinge on the destabilization of memory, as well as its reconsolidation. With this in mind, one possibility is that, since the infusions were given prior to reactivation, all infusions except the combined AP-5/SCH23390 inhibited memory destabilization, thus preventing reconsolidation from occurring; antagonism at both NMDARs (Ben Mamou et al., 2006; Milton et al., 2013) and DR1s (Rossato et al., 2014) has been known to inhibit destabilization. Alternatively, the effect of the combined AP-5/SCH23390 infusion to impair pavlovian memory reconsolidation may, in fact, also reflect an enhancement of pavlovian memory destabilization during the reactivation session, enabling its reconsolidation to be disrupted; infusions of both AP-5 (Milton et al., 2008a; Wu et al., 2012) and SCH23390 (Maroun and Akirav, 2009) are known to impair reconsolidation.

While there is no independent way to assess successful memory destabilization, other than the reconsolidation impairment itself, the acute effect of combined AP-5/SCH23390 during reactivation may be consistent with an enhancement of reactivation-induced destabilization. Notably, the acute effect was only observed with the combined infusion. Moreover, that the acute effect constituted a performance deficit is not inconsistent with facilitated destabilization, as successful memory expression is not required for destabilization (Ben Mamou et al., 2006; Rodriguez-Ortiz et al., 2012; Milton et al., 2013; Lee and Flavell, 2014). Finally, in a pavlovian contextual fear setting, it has recently been demonstrated that pharmacological treatment can stimulate memory destabilization under behavioral conditions that, by themselves, are ineffective (Lee and Flavell, 2014). Whether or not the hypothetical enhancement of pavlovian memory destabilization by AP-5/SCH23390 has any impact on instrumental memory destabilization cannot be determined from our results. This, therefore, leaves open the question of whether two independent memory traces can be simultaneously destabilized.

While enhanced destabilization by intra-accumbal AP-5/SCH23390 is consistent with the behavioral data, an obvious weakness of the argument is a lack of mechanistic rationale. It is well established that local NMDAR activity is required for the reconsolidation of appetitive (Milton et al., 2008a; Wu et al., 2012) and aversive (Campeau et al., 1992; Rodrigues et al., 2001; Goosens and Maren, 2004; Lee and Hynds, 2013; Milton et al., 2013) pavlovian memories. Furthermore, existing evidence indicates that that antagonism of NMDARs blocks, rather than enhances, destabilization (Ben Mamou et al., 2006; Milton et al., 2013). While there is less evidence that D1R antagonism impairs reconsolidation (Sherry et al., 2005; Diergaarde et al., 2008; Maroun and Akirav, 2009), there is also a report of D1R antagonism disrupting the destabilization of object recognition memory (Rossato et al., 2014). Additionally, dysregulation of dopaminergic midbrain neurons prevents appetitive memory destabilization (Reichelt et al., 2013). Therefore, within such a literature and framework, there is little reason to suggest that antagonism of NMDARs or D1Rs might enhance memory destabilization. That said, infusion of AP-5 and SCH23390 individually did not result in the putative enhancement of memory destabilization.

While speculative, our data may imply that combined, but not individual, antagonism of D1Rs and NMDARs enhances destabilization of appetitive pavlovian memory, rather than preventing it. A growing body of evidence suggests NMDARs functionally interact with G-protein-coupled receptors, like D1Rs (for review, see Fan et al., 2014). While some of this interaction is mediated by convergent signaling pathways, there also appears to be a subunit-specific direct protein−protein interaction between D1Rs and NMDARs (Lee et al., 2002; Pei et al., 2004). Interestingly, activation of the D1R can facilitate binding of the calcium-sensor calmodulin (CaM) to the NR1 subunit of the NMDAR (Lee and Liu, 2004), leading to activation of a variety of downstream signaling molecules, including CaM-dependent kinase II (CaMKII). Notably, CaMKII has been implicated in the induction of both long-term potentiation and depression; the switch between the two appears to be determined by the precise phosphorylation state of specific sites (Pi et al., 2010), affecting the substrate selection of CamKII (Coultrap et al., 2014). Importantly, CaMKII also appears to be important in recruitment of the proteasome pathway (Bingol et al., 2010), which is critical in memory destabilization (Lee et al., 2008). By appealing to such a literature, we can speculate that co-antagonism of D1Rs and NMDARs may have biased intracellular signaling pathways, such as CaMKII, towards conditions that favor protein degradation and memory destabilization. Given that many cellular pathways involved in consolidation, destabilization, and reconsolidation appear to be shared, investigation of how specific surface-receptor subunits interact may prove a fruitful avenue to explore to understand how these pathways diverge.

Returning to the systemic MK-801 experiments, it is notable that similar effects were observed in both the sucrose and cocaine settings. In previous studies of pavlovian memory reconsolidation, important differences have been observed in the reconsolidation of cue–sucrose and cue–cocaine memories. For example, the cue–cocaine memory was more easily destabilized by noncontingent cue exposure than was the cue–sucrose memory (Lee et al., 2006a; Lee and Everitt, 2008a). Here, it is not clear whether sucrose and cocaine instrumental memories have identical destabilization parameters. The common capacity of the VR5 reactivation to destabilize the instrumental memory may, in fact, reflect differential parameters, as the acquisition data were clearly different between the two experiments. The mean number of total sucrose reinforcements across all experiments was 71.0, compared with 37.7 in the cocaine experiment. Therefore, it is possible that with matched training conditions, instrumental memories for sucrose and cocaine reinforcement may require different reactivation parameters for successful destabilization. Nevertheless, the fundamental conclusion that instrumental cocaine memories can be disrupted by targeting their reconsolidation has potential value for translational exploitation in the treatment of compulsive cocaine-seeking behavior. It remains to be determined, however, whether the reconsolidation of well-learned instrumental cocaine memories can be disrupted, as has previously been demonstrated for sucrose (Exton-McGuinness et al., 2014b).

In summary, our results demonstrate that weakly-trained instrumental memories for both sucrose and cocaine reinforcement do destabilize following a shift to a variable ratio schedule, and their reconsolidation can be disrupted by systemic MK-801. Interestingly, the NAc does not appear to be a central locus of action for MK-801; however, coactivation of D1Rs and NMDARs in the NAc may play a role in both the destabilization and reconsolidation of the pavlovian components associated with lever-pressing behavior. Importantly, our data provide evidence that instrumental memory reconsolidation can be disrupted to diminish cocaine seeking. This provides strong support for the viability of novel reconsolidation-based therapies to diminish maladaptive behaviors such as drug addiction.

## Synthesis

The decision was a result of the Reviewing Editor Howard Eichenbaum and the peer reviewers coming together and discussing their recommendations until a consensus was reached. A fact-based synthesis statement explaining their decision and outlining what is needed to prepare a revision is listed below. The following reviewer agreed to reveal his identity: Karim Nader

The aim of this paper was to investigate whether an instrumental memory that has been formed following weak training undergoes reconsolidation. This is novel and important because up until recently it was not clear if instrumental memories undergo reconsolidation at all. Some evidence indicates that well trained instrumental memories could destabilize and reconsolidate, but these memories are fundamentally different from those formed following weak training. In a series of well-designed experiments, the authors show that systemic administration of MK-801, an NMDA receptor antagonist, disrupted the reconsolidation of lever pressing for sucrose and cocaine. This finding was unique to the group that was reminded of the memory, and was not found when the drug was administered without memory reactivation, when the memory was briefly reactivated, reactivated without reward, or reactivated without the drug. The effect also did not extend to instrumental performance of nosepoking within group. By using intra-cerebral injections, the authors further showed that the locus of MK-801 interference was probably not the nucleus accumbens, but co-activation of NMDA and Dopamine D1 receptors in the nucleus accumbens appear to have a role in reconsolidation.

Overall, this is an interesting study with rigorous methods. Understanding the boundary conditions of instrumental reconsolidation driven by rewards has important implications for relapse and drug-seeking behavior. Although this study could be of wide interest to the fields of learning and memory and drug addiction, the paper seems to be written for specialists only. Many aspects of the study related to the hypotheses and manipulations become clear only later in the discussion, and the result section is written as a list. These issues could be fixed by extensive re-writing of the manuscript.

The main issues that should be clarified upfront are:

– Destabilization and reconsolidation are separate processes. Here, destabilization could only be indirectly inferred from reconsolidation blockade. This dissociation becomes evident when interpreting the results of the various control conditions and drug effects. It is therefore important that the authors clarify these terms and their indication in the current protocol.

– Relevant to destabilization is that fact that the drugs were infused prior to reactivation, which is a limitation of the experimental design, this should be clarified.

– The difference between lever pressing and nosepoking and why the latter serves as control should be clarified before we see nosepoking results.

– VR5 reinforcement schedule is mentioned as being previously successful in reactivating instrumental memory, but there is no explanation of why and what does it mean.

– Also terms such as "conditioned motivation," "extinction," and VR5-induced "new learning" appear in discussion without explanation.

– The requirement for short-term memory test is explained after discussing the results of this test.

– Large parts of the discussion are dedicated to the question of whether the Pavlovian or instrumental components of the memory were disrupted. This is a very important distinction but there is no explanation of what these components actually are in the lever-pressing protocol.

– Note that "intra-accumbal MK-801" is mentioned several times instead of AP-5.

– One of the main differences between reward and punishment learning is that reward learning typically involved extensive training whereas aversive learning is very rapid. The use of weak reward training here provides a great opportunity for the authors to discuss their findings in the context of reconsolidation of fear learning.

## Author Response

The aim of this paper was to investigate whether an instrumental memory that has been formed following weak training undergoes reconsolidation. This is novel and important because up until recently it was not clear if instrumental memories undergo reconsolidation at all. Some evidence indicates that well trained instrumental memories could destabilize and reconsolidate, but these memories are fundamentally different from those formed following weak training. In a series of well-designed experiments, the authors show that systemic administration of MK-801, an NMDA receptor antagonist, disrupted the reconsolidation of lever pressing for sucrose and cocaine. This finding was unique to the group that was reminded of the memory, and was not found when the drug was administered without memory reactivation, when the memory was briefly reactivated, reactivated without reward, or reactivated without the drug. The effect also did not extend to instrumental performance of nosepoking within group. By using intra-cerebral injections, the authors further showed that the locus of MK-801 interference was probably not the nucleus accumbens, but co-activation of NMDA and Dopamine D1 receptors in the nucleus accumbens appear to have a role in reconsolidation.

Overall, this is an interesting study with rigorous methods. Understanding the boundary conditions of instrumental reconsolidation driven by rewards has important implications for relapse and drug-seeking behavior. Although this study could be of wide interest to the fields of learning and memory and drug addiction, the paper seems to be written for specialists only. Many aspects of the study related to the hypotheses and manipulations become clear only later in the discussion, and the result section is written as a list. These issues could be fixed by extensive re-writing of the manuscript.

We thank the reviewers for their helpful comments, and we have re-written the manuscript to address their concerns. In particular we have overhauled our results section to highlight the relevance of each analysis and make our paper more accessible to a non-specialist reader. Individual points are addressed below.

The main issues that should be clarified upfront are:

– Destabilization and reconsolidation are separate processes. Here, destabilization could only be indirectly inferred from reconsolidation blockade. This dissociation becomes evident when interpreting the results of the various control conditions and drug effects. It is therefore important that the authors clarify these terms and their indication in the current protocol.

The introduction has been edited to make this distinction more clear. We have also added text to our methods, results section and discussion to make this distinction explicit, and aid the reader in interpreting our findings.

– Relevant to destabilization is that fact that the drugs were infused prior to reactivation, which is a limitation of the experimental design, this should be clarified.

We have highlighted this limitation to the reader in our discussion.

– The difference between lever pressing and nosepoking and why the latter serves as control should be clarified before we see nosepoking results.

The results section has been re-written to address the importance of why each analysis was conducted. The relevance of the nosepoke analysis has been included in both the method and results sections, prior to presentation of the first nosepoke analysis, to aid the reader.

– VR5 reinforcement schedule is mentioned as being previously successful in reactivating instrumental memory, but there is no explanation of why and what does it mean.

We have provided some additional discussion around how the variability of the VR5 reactivation may have contributed to its potency to destabilize instrumental memory, and why this feature may be important, specifically in comparison with the FR1 reactivation. However, the precise reason for the success of the VR5 reactivation is unclear. As we point out when discussing the results of the cocaine study, the cocaine- and sucrose-reinforced memories are not as equivalent as they may seemingly appear at first glance. As such there may have been an element of luck that the VR5 was successful in both cases.

– Also terms such as "conditioned motivation," "extinction," and VR5-induced "new learning" appear in discussion without explanation.

Additional text has been added to the discussion to clarify the definition of these terms where they appear. The definition of extinction within the context of our experiments has also been made explicit with additional text throughout the manuscript. We have also changed VR5-induced “new learning” to read “additional learning” to clarify that by this we mean strengthening of the existing memory, whether that be by reconsolidation-mediated updating or formation of a new memory.

– The requirement for short-term memory test is explained after discussing the results of this test.

We have added text to the methods and results sections to highlight the relevance of the pr-STM test, prior to presentation of the results.

– Large parts of the discussion are dedicated to the question of whether the Pavlovian or instrumental components of the memory were disrupted. This is a very important distinction but there is no explanation of what these components actually are in the lever-pressing protocol.

We have expanded this section of the discussion to include a brief description of the pavlovian and instrumental contributions to lever pressing, prior to the core of the discussion on pavlovian vs instrumental components of lever pressing memory.

– Note that "intra-accumbal MK-801" is mentioned several times instead of AP-5.

MK-801 was also infused intra-accumbally, as well as AP-5 (Figure 6). We have edited the methods and results section to make this clearer.

– One of the main differences between reward and punishment learning is that reward learning typically involved extensive training whereas aversive learning is very rapid. The use of weak reward training here provides a great opportunity for the authors to discuss their findings in the context of reconsolidation of fear learning.

This is a very interesting point, however we do not feel a broader discussion of fear literature would enhance our manuscript. Fear conditioning is often achieved in a single learning trial, and it is clear that our data cannot easily be compared given the significantly larger number of reinforcements (essentially action-reward pairings) used in our study compared to the fear literature. While there is an interesting discussion to be had regarding the age, rather than putative strength, of our appetitive memory vs aversive one or two-trial learning (typically spread over 1-2 days), there is a paucity of literature on the temporal boundary conditions which constrain reconsolidation. Such a discussion would be beyond the scope of our study and require additional data to be collected in order for us to provide any meaningful insight (see Suzuki et al. 2004 for example).
